# Democratic quality and excess mortality during the COVID-19 pandemic

**DOI:** 10.1038/s41598-024-55523-6

**Published:** 2024-04-04

**Authors:** José-Jesús Martín-Martín, Manuel Correa, Araceli-María Rojo-Gallego-Burín, María-Teresa Sánchez-Martínez, Luisa Delgado-Márquez, María-Ángeles Ortega-Almón

**Affiliations:** 1 Department of Applied Economics, University of Granada, University Campus of Cartuja s/n, 18008 Granada, Spain; 2 Department of Business Management, University of Granada, Granada, Spain

**Keywords:** COVID-19, Coronavirus, Excess mortality, Democracy index, Democratic quality, Efficient autocracies, Diseases, Risk factors

## Abstract

The aim of this study is to analyse the relationship between democratic quality and excess mortality produced in the year 2020 before COVID-19 vaccinations were generalised. Using cross-sectional data from 80 countries on five continents, multiple linear regression models between excess mortality, the general democracy index and its disaggregation into five categories: electoral process and pluralism, government functioning, political participation, political culture and civil liberties were estimated. The analysis also considered, public health spending per capita, overweight inhabitants, the average temperature of the country, population over 65 years of age, The KOF Globalisation Index, and the Gross National Income per capita as control variables. It was possible to establish a strong inverse association between excess mortality per million inhabitants and the general democracy index and four of its five categories. There was a particularly strong relationship between excess mortality and the political culture dimension (−326.50, p < 0.001). The results suggest that the higher the democratic quality of the political institutions of a State and particularly of their political culture the more improved the response and management of the pandemic was in preventing deaths and protecting their citizens more effectively. Conversely, countries with lower democracy index values have higher excess mortality. Quality democratic political institutions provide more effective public health policies in the face of the COVID-19 pandemic.

## Introduction

Severe acute respiratory syndrome coronavirus 2 (SARS-CoV-2) has caused a global health and social crisis, which has disrupted lives and spread a wave of suffering and anxiety unprecedented in recent times. By the end of January 2022, there had been 399,600,607 confirmed cases and 5,757,562 deaths from COVID-19^1,2^. The response of countries, prior to the availability of vaccines, consisted of containment measures, reduced mobility, and transmission chain tracing. However, the effectiveness of these measures is conditioned by the institutional and political characteristics of each individual country^3^.

Recent research has explored the relationship between economic, social and political characteristics of countries and their degree of effectiveness in dealing with the pandemic^4,5^. In a context of increasing geopolitical polarisation between autocracies and democracies and a general decline in democratic quality^6,7^ a crucial aspect of this debate is to specifically analyse the relationship between democratic quality of countries and their degree of success in coping with COVID-19. Several research papers postulate that autocracies have been more efficient due to their greater speed and capacity to impose measures which limit freedoms and involve social distancing^8,9^. Engler et al.^10^ suggest that democratic principles make governments reluctant to implement mandatory health policies. The higher the levels of democratic quality the lower the restrictions on citizens' freedoms and national public health policies, leading to higher levels of transmission, infections and deaths^10,11^.

The aim of this paper is to analyse the relationship between a country’s democratic quality and their success in their fight against COVID-19 during the year 2020 before large-scale implementation, at least in developed countries, of vaccines. The results are relevant to the current debate on the effectiveness and resilience of democracies versus authoritarian regimes when coping with external global shocks such as pandemics and perhaps other potential catastrophes arising from the technological revolution, globalisation and climate change. A crucial debate which, although it inevitably involves confronting intrinsically normative values, must be conducted with the best available evidence.

The hypothesis of this study is that democratic quality is associated with better pandemic management, resulting in fewer deaths, if a sufficient time interval is considered and a more comprehensive measurement is used than COVID-19 associated mortality. Democracies compensate for the rapid decision-making processes of autocracies if the complex formal and informal institutional networks which shape them function properly and provide legitimacy, transparency and broad social consensus towards the adopted measures.

Although democracy is currently the political system in the world by majority, at 59%, compared to 13% autocracies with the remaining 28% sharing both democratic and autocratic elements^12^, the decline in the democratic quality of countries is highlighted by various studies. According to International Institute for Democracy and Electoral Assistance^6^ the pandemic accelerated a trend started in 2015 of the quality of democratic institutions backsliding. In 2020, the number of countries which had regressed democratically surpassed those which had advanced. In 2020, for the first time since 2010, the Democracy Index scores produced by The Economist Intelligence Unit^7^ decreased in most countries, 116 out of 167 (almost 70%), which meant a decline in the overall score compared to 2019 was registered. The overall average score fell from 5.44 in 2019 to 5.37 in 2020, the worst result since 2006, when the index was first compiled.

This study uses the general democracy index and its five categories or dimensions developed by The Economist Intelligence Unit as a measure of democratic quality. The five categories being, electoral process and pluralism (category I), government functioning (category II), political participation (category III), political culture (category IV) and civil liberties (category V). Each dimension or category is in turn composed of between eight and seventeen indicators (“Annex I”). The five dimensions are interrelated and form a coherent conceptual set^7^. The democratic quality of a country cannot be solely based on political and civil liberties although it is fundamental; it must also consider a government's capacity to implement policies and understand informal institutions which are key to the proper functioning of the formal institutional architecture of the state, such as participation and political culture. Democracies are strong when citizens participate and are actively involved in political debate. Likewise, a strong democracy is one whose consensus on the functioning of democracy is high and speculatively strong in its rejection of other forms of government^7^.

Most studies have used the daily average number of COVID-19 cases and deaths per million inhabitants or the infection rate^5,13,14^ to measure the impact of the pandemic.

Excess mortality defined by the World Health Organization^15^ as mortality above that which would be expected based on the non-crisis mortality rate of the population of interest has been used in this research. Excess mortality is thus mortality which is attributable to the crisis conditions. It can be expressed as a rate (the difference between observed rates and non-crisis mortality rates), or as a total number of excess deaths".

Excess mortality captures more comprehensively the health impact of the pandemic, since it reports not only deaths confirmed by COVID-19, but also deaths not correctly diagnosed and registered, as well as deaths from other causes which are attributable to the general conditions of the crisis, thus constituting a more reliable metric for comparing countries^16^.

Several recent works support the use of excess mortality, both in analyses applied to a single country or territory^17–20^, and for international comparisons^21–23^ in which they contrast its greater suitability for identifying the number of deaths resulting from COVID-19. Similarly, Karlinsky and Kobak^24^, consider excess mortality a more objective indicator of the number of COVID-19 deaths, having collected and analysed weekly, monthly or quarterly all-cause mortality data from 103 countries and territories.

The limited but growing literature on the relationship between the quality of democracy and level of performance of pandemic management can be grouped into the categories proposed by Cassan and Steenvoort^25^ "efficient autocracies hypothesis" and "biased autocracies hypothesis".

Most studies fall onto the side of the efficient autocracies hypothesis, which assert the superiority and greater efficiency of autocracies, due to the difficulties faced by democracies when implementing policies restricting freedoms such as confinement or social distancing, which impact on a lower rate of contagion and mortality^8,26–29^.

Dempere^13^, through a sample of 156 countries, finds that countries with the highest democracy indices applied the mildest social restrictions to control the first wave of the COVID-19 outbreak, as measured by the average daily stringency index. These countries suffered a more severe impact of the pandemic as confirmed by the highest daily cases averages, deaths per million and the highest mortality rate. Yao et al.^14^ examined the association between the Democracy Index and the COVID-19 case fatality rate in a sample of 148 countries. Their results establish that the higher the Democracy Index the more COVID-19 deaths there were in the early stage of the pandemic. Valev^5^ analysed the statistical relationships of total COVID-19 cases and deaths per million inhabitants in 45 countries, identifying correlations between the rate of infections and deaths per million with the Democracy Index.

The "biased autocracies hypothesis" argues that a possible explanatory reason for the lower infection rates and case fatality rate is due to manipulation of COVID-19 infection and mortality rate records by autocracies, which created systematic differences between reported infection and mortality rates and actual infection and mortality rates. Work by Annaka^30^, Kapoor et al.^31^, Adiguzel et al. (2020)^32^ and Cassan and Steenvoort^25^, among others, postulate that autocracies' lower levels of contagion and lethality are a product of opacity, lack of transparency and data manipulation.

A third hypothesis in addition to the two discussed above is the "simply different autocracies hypothesis" which emphasises the structurally different characteristics such as younger population or lower testing capacity of democracies compared with autocracies which led to different outcomes in pandemic management. Ashraf^33^, using COVID-19 deaths data from 120 countries, finds little evidence of a relationship between democracies and COVID-19 deaths, however, they do identify robust correlations for countries with higher levels of the population aged 14–65 years and lower COVID-19 mortality rates. The three hypotheses are not necessarily incompatible with each other, and it is the task of research to elucidate the degree of evidence for each.

This paper makes two contributions to the literature. First, it estimates the relationship between excess mortality and the democratic quality of countries. Second, in addition to the general democracy index, five specific dimensions of democratic quality are considered, allowing for scrutiny of the relationship between COVID-19 deaths and democratic quality. This is the first work which combines excess mortality as a measurement of the impact of the Coronavirus and a multidimensional approach to democratic quality.

## Methods

The analysis was carried out across 80 countries on five continents for the four categories established by the general Democracy Index "full democracy" (21), "imperfect democracy" (38), "hybrid regime" (11) or "authoritarian regime" (10). “Annex II” shows the values of the excess mortality and general democracy index for each country and each of the five categories of democracy used.

The impact of the pandemic is measured by excess mortality per million inhabitants, and the variables of interest are the general democracy index and its 5 categories as a proxy for the democratic quality of the countries. As control variables we have included those which the research has identified as associated with COVID-19 mortality, in particular, per capita income^34^, health expenditure^35,36^, KOF Globalisation index^37^, temperature^38–40^, population aging^41^, and obesity^42,43^. Table 1 shows the definition and source of information for the variables used in the analysis.

**Table 1:** Variables for excess mortality for COVID-19 analysis.

Variable	Repository	Variable description
Excess mortality per million inhabitants	Our world in data https://ourworldindata.org/excess-mortality-covid Maps available at https://ourworldindata.org/grapher/excess-mortality-p-scores-projected-baseline?tab=map&time=2020-12-31	Gross quantity of observed deaths on 31 December 2020, by subtracting the average number of deaths, on that date, with respect to the deaths of the previous five years
Overall democracy index	The Economist Intelligence Unit Limited (2021)	Average value of the different categories of democracy
Democracy category 1 Electoral process and pluralism		Drawn from the 12 different items related to the Electoral Process and Pluralism
Democracy category 2 Government function		Drawn from the 14 different items related to the functioning of government in category II
Democracy category 3 political participation		Based on the 9 items in category III
Democracy category 4 Democratic political culture		Based on the 8 items in category IV
Democracy Category 5 Political freedoms		Based on the 17 items in category V
GNI per capita	World Bank. Data. GNI per capita. Atlas Method: https://datos.bancomundial.org/indicator/NY.GNP.PCAP.CD	Gross National Income per capita converted to USD ($) using the World Bank Atlas method, divided by mid-year population
Public health expenditure per capita	World Health Organization^2^. Global Health Expenditure Database: https://apps.who.int/nha/database	Public heath expenditure from domestic sources per capita expressed in international dollars at purchasing power parity
KOF Globalisation index	KOF Swiss Economic Institute https://kof.ethz.ch/en/forecasts-and-indicators/indicators/kof-globalisation-index.html	KOF Globalisation index for 2020
Average temperature	World Bank https://climateknowledgeportal.worldbank.org/	Annual mean average monthly temperature recorded in the year 2020
Over 65s (as a % of the population)	Our world in Data https://ourworldindata.org/search?q=Population+by+age+group	Percentage of population over 65 years of age out of the total population
Overweight (as a % of the population)	Our world in Data https://ourworldindata.com	Percentage of adult population (18 years and older) with a body mass index between 25 and 30 (BMI = weight [kg]/height [m2])

Author’s own based on Our World in Data (2022) https://ourworldindata.org/, The Economist Intelligence Unit Limited^7^. Democracy Index 2020. In sickness and in health? Available at https://www.eiu.com/n/campaigns/democracy-index-2020/, World Health Organization^2^. Global Health Expenditure Database. Available at: https://apps.who.int/nha/database, World Bank. Data. GNI per capita. Atlas Method. Available at: https://datos.bancomundial.org/indicator/NY.GNP.PCAP.CD, and Climate Change Knowledge Portal. Available at: https://climateknowledgeportal.worldbank.org/, and KOF Globalisation index. Available at: https://kof.ethz.ch/en/forecasts-and-indicators/indicators/kof-globalisation-index.html.

*GNI* gross national income, *USA* United States of America.

The data corresponds to the year 2020, except for excess mortality, which corresponds to December 31, 2020, percentage of overweight inhabitants, from the year 2016, health expenditure, from the year 2018 and Gross National Income from 2019.

The dependent variable is the excess mortality (ED) per million population, as the gross number of deaths observed on December 31, 2020, subtracting an estimate of the expected deaths (average deaths), at that date with respect to the deaths of the previous 5 years (Our World in Data, 2022)^44^.

$${\text{Excess \, Deaths}}^{2020\, \mathrm{ per \, millon}}=\frac{ {\mathrm{Reported \, Deaths}}^{2020}-{\mathrm{Expected \, Deaths}}^{(2019-2014)}}{\mathrm{1.000.000}}$$

The Democracy Index, prepared by The Economist, (uses a scale of 0 to 10 based on ratings of 60 indicators, grouped into five categories^7^). As mentioned above, “Annex I” lists the items rated in each category or dimension. The overall index is the simple average of the indices of the five categories.

Based on the scores in each dimension, each country is in turn classified as one of the following four regime types: (a) "full democracy" (score > 8), (b) "imperfect democracy" (> 6 score ≤ 8), (c) "hybrid regime" (> 4 score ≤ 6) or (d) "authoritarian regime" (score ≤ 4).

Descriptive analysis, correlation analysis and multiple linear regression analysis were performed. For the correlation analysis between the dependent variable and the rest of the variables, the Shapiro–Wilk test was used to identify the distribution of the variables and Pearson's and Spearman's correlation coefficients were calculated. Six multiple linear regression models have been estimated, one with the general democracy index, and five more with the different categories. Analytically:

$${\text{Y}}_{{\text{i}}} = {\beta_{0}} + {\beta}_{{1}} {\text{X}}_{{{\text{1i}}}} + {\beta}_{{2}} {\text{X}}_{{{\text{2i}}}} + {\beta}_{{3}} {\text{X}}_{{{\text{3i}}}} + \ldots \, + {\beta}_{{\text{n}}} {\text{X}}_{{{\text{ni}}}} + {\text{e}}_{{\text{i}}}$$

where Y_i_ is the excess mortality per million inhabitants for each country considered, X_ni_ is the set of independent variables included in the models for each country, β_i_ are the coefficients and e_i_ is the error term. Several tests were performed to verify the consistency of the estimates and the specificity of the model. The analyses were carried out with Stata^©^ 15.1.

The authors confirm that all methods were performed in accordance with the relevant guidelines and regulations of *Scientific Reports*.

## Results

Table 2 summary of descriptive statistics of variables used.

**Table 2:** Descriptive statistics of variables to analyse excess mortality by COVID-19 and democracy.

Variable	Mean	Std. dev	Min	Max
Excess mortality per million inhabitants	923.02	879.69	-658.00	3377.00
Overall democracy index	6.68	1.89	2.12	9.81
Category I (electoral process y pluralism)	7.77	3.02	0.00	10.00
Category II (government function)	6.02	2.05	1.50	9.64
Category III (political participation)	6.50	1.58	2.78	10.00
Category IV (political democratic culture)	6.06	2.00	3.13	10.00
Category V (civil liberties)	7.03	2.11	0.88	9.71
GNI per capita	23,019.01	21,950.55	1240.00	87,950.00
Health expenditure per capita	1691.24	1496.45	111.33	5817.63
KOF global index	74.34	9.91	51	91
Average temperature	14.67	7.74	-4.35	28.48
Over 65s (as % of the population)	14.28	6.19	1.69	28.40
Overweight (as % of the population)	58.23	9.37	26.1	70.3

80 countries. 2020.

Author’s own based on Our World in Data (2022) https://ourworldindata.org/, The Economist Intelligence Unit Limited^7^. Democracy Index 2020. In sickness and in health? Available at https://www.eiu.com/n/campaigns/democracy-index-2020/, World Health Organization^2^. Global Health Expenditure Database. Available at: https://apps.who.int/nha/database, World Bank. Data. GNI per capita. Atlas Method. Available at: https://datos.bancomundial.org/indicator/NY.GNP.PCAP.CD, and Climate Change Knowledge Portal. Available at: https://climateknowledgeportal.worldbank.org/, and KOF Globalisation index. Available at: https://kof.ethz.ch/en/forecasts-and-indicators/indicators/kof-globalisation-index.html.

*Std. dev*. standard deviation. Calculations performed through STATA^©^ 15.1

The average excess mortality per million inhabitants is 923 deaths per million inhabitants, with a minimum of -658 (Uruguay) and a maximum of 3377 (Armenia). The average of the general democracy index and the 5 categories corresponding to the different dimensions take values between 6 and 7, being slightly higher in categories I and V. The mean average of the general democracy index is 6.68 with a minimum of 2.12 for Uzbekistan and a maximum of 9.81 for Norway.

The correlation study of excess mortality per million inhabitants and the rest of the variables are shown in Table 3.

**Table 3:** Correlations between excess COVID-19 mortality and general and democracy-specific indices.

Variable	Type of correlation	Coefficient	P-value
Overall democracy Indices	Spearman	−0.45	0.00
Category I (electoral process and pluralism)	Spearman	−0.34	0.00
Category II (government function)	Pearson	−0.46	0.00
Category III (political participation)	Pearson	−0.24	0.00
Category IV (democratic political culture)	Pearson	−0.54	0.00
Category V (civil liberties)	Spearman	−0.37	0.00
GNI per capita	Spearman	−0.31	0.00
Health expenditure per capita	Spearman	−0.24	0.03
KOF global index	Spearman	−0.14	0.21
Average temperature	Spearman	−0.21	0.06
Over 65s (as % of population)	Spearman	0.09	0.41
Overweight (as % of population)	Spearman	0.10	0.35

80 countries. 2020.

Calculations performed through con STATA^©^ 15.1

Authors own using Our World in Data (2022) https://ourworldindata.org/, The Economist Intelligence Unit Limited^7^. Democracy Index 2020. In sickness and in health? https://www.eiu.com/n/campaigns/democracy-index-2020/, World Health Organization^2^. Global Health Expenditure Database: https://apps.who.int/nha/database. World Bank. Data. GNI per capita. Atlas Method: https://datos.bancomundial.org/indicator/NY.GNP.PCAP.CD. KOF Globalisation index. Available at: https://kof.ethz.ch/en/forecasts-and-indicators/indicators/kof-globalisation-index.html.

The results show a significant correlation (p-value < 0.05) and inverse correlation between excess mortality per million inhabitants and the overall democracy index and 4 of the different categories, political participation is not significant. There is also a correlation between public health expenditure per capita, gross national income and mean temperature (p-value < 0.06). The correlation coefficient for political culture is the highest value, −0.54, suggesting a strong negative association with excess mortality per million inhabitants. Higher values of democracy indices are therefore related to lower levels of COVID-19 deaths.

The results of the six multiple linear regression models corresponding to the general democracy index and to each of the five different categories are shown in Table 4. The Breusch-Pagan test was performed, and all models showed homoscedasticity, except category III model (whose coefficient is not significant); the estimators are therefore efficient as well as linear, unbiased and consistent. The possible collinearity of the independent variables in the different models was tested using the variance inflation factor. The results by variables showed VIF values lower than 10, and the mean value in no model was higher than 5.

**Table 4:** Results of multivariate regression models in the analysis of excess mortality per million population and democratic quality.

Variable	Model 0	Model 1	Model 2	Model 3	Model 4	Model 5
	Coefficients (p-value *)					
Overall democracy indices	−222.27***					
Category I (electoral process and pluralism)		−75.64**				
Category II (government function)			−203.14***			
Category III (political participation)				−91.02		
Category IV (democratic political culture)					−327.41***	
Category V (civil liberties)						−149.23***
GNI per capita	−0.00	−0.01	−0.00	−0.00	0.00	−0.00
KOF global index	7.14	1.60	9.10	−3.49	1.86	−2.21
Health expenditure per capita	−0.09	−0.15	−0.07	−0.13	−0.08	−0.12
Average temperature	−21.16*	−23.46*	−20.65	−29.32*	−19.66*	−21.08
Over 65s (as a % of the population)	44.20*	42.43*	33.03	29.61	41.86**	48.90**
Overweight (as a % of the population)	22.54**	24.75**	18.13*	25.69***	22.38***	25.56***
Constant	574.52	199.89	509.65	731.69	1205.90	650.11
R^2^	0.4224	0.3637	0.4063	0.3375	0.5332	0.3901

80 countries. 2020.

Calculations performed using STATA^©^ 15.1. P-values expressed as: ***p < 0.01, **p < 0.05, *p < 0.10.

Authors own using Our World in Data (2022) https://ourworldindata.org/, The Economist Intelligence Unit Limited^7^. Democracy Index 2020. In sickness and in health? https://www.eiu.com/n/campaigns/democracy-index-2020/, World Health Organization^2^. Global Health Expenditure Database.: https://apps.who.int/nha/database. World Bank. Data. GNI per capita. Atlas Method: https://datos.bancomundial.org/indicator/NY.GNP.PCAP.CD.

The coefficient between excess mortality per million inhabitants and the general democracy index is significant and negative, i.e., on average, an increase or improvement of this index by one unit means a decrease of 222 deaths per million inhabitants.

The coefficients of categories I and II, which measure "Electoral process and pluralism" and "Government function" respectively, are significant and negative. A one-point increase in these indices is associated with average reductions of 75 and 203 deaths per million inhabitants, respectively. The coefficient for "Political participation" (Category III), although negative, is not statistically significant. Finally, categories IV and V "Political and democratic culture" and "Civil Liberties" show a significant association with excess mortality per million inhabitants. “Political and democratic culture" strongly correlates with the dependent variable; an increase of one point in this category would mean an average decrease of 327 deaths per million inhabitants, a remarkable figure, given that it is one third of the mean average and standard deviation of this variable. Similarly, category V, "Civil liberties" is associated with a reduction of 149 deaths for each point increase in the value of this category on average.

The results of the rest of the variables analysed show that neither the coefficient of public health expenditure per capita nor that of GNI per capita are statistically significant (the inclusion of this variable in its quadratic form was alternatively tested, ruling out a possible non-linear relationship with the dependent variable). nor that of KOF globalisation index. On the other hand, the coefficients of the overweight percentage of the population, the average temperature in each country and the percentage of people over 65 are significant and with the expected sign. There is a direct association between overweight people and people over 65, and an inverse association with the average temperature.

The coefficients of determination of the six models are above 0.33, model 4 specifically stands out with a value of 0.53.

## Conclusion and discussion

The democratic quality of a country is related to a better response to the COVID-19 pandemic by way of lower excess mortality. Both the overall democracy index and four of its component categories show a robust inverse association with direct and indirect deaths caused by COVID-19. This result is at odds with work categorised by the efficient autocracies hypothesis which indicates that the speed of decision-making to deal with the pandemic coupled with the coercive power to implement measures of confinement and restriction of civil liberties without debate and opposition is more effective than deliberative and reluctancy to restrict liberties models characteristic of democracies^5,13,14,26^. The fundamental reason for this disparity is likely the different counting metric for the number of COVID-19 fatalities. Excess mortality is a more comprehensive and complete metric than the mortality rate, incorporating not only diagnosed COVID-19 cases but also deaths recorded not as COVID-19 deaths, but which are a direct or indirect consequence of the pandemic.

Some recent studies, with a different approach, present results with some similarity to those of this study. Bayerlein et al.^45^ with a sample of 42 countries study the effect of "populist" governments on excess mortality, concluding that excess mortality in countries with populist governments exceeds excess mortality in non-populist countries by 8 percentage points. Charron et al.^46^, in their analysis on 153 European regions, find evidence that ideological polarisation of citizens is associated with higher excess mortality.

The results of this study are strengthened by if also framed within the "biased autocracies hypothesis". Annaka^30^ analysed the relationship between political regimes, data transparency and COVID-19 fatalities using cross-national data from over 108 countries, their results point to possible data manipulation, rather than the nature of political regime characteristics per se, as the most significant source of low fatality rates in authoritarian countries. Cassan and Steenvoort^25^ with a sample of 137 countries, estimate that if all countries analysed in their model had been fully democratic, the number of deaths reported in the first year of the pandemic would have increased by approximately 400,000 (or 13% of deaths at that time). Assuming this hypothesis, it is plausible to argue that the excess mortality data is also underestimated in countries with lower overall democracy indices, i.e., hybrid and authoritarian regimes, and consequently their values would be higher than those reported. From this perspective, the estimates of this study can be considered a baseline, which could only be improved if "real" values were recorded for countries with lower democratic quality.

One relevant finding of this study is the significant relationship between excess mortality and four of the five categories which make up the overall democracy index. The relationship between category IV (democratic political culture) and excess mortality (−327.41) is particularly robust. Its value which aggregates the eight items (“Annex I”) is very stable over time and full democracies present high values in this category. Only one country is below 7.5 Austria (6.88)^7^. It essentially expresses the degree of legitimacy in democratic institutions on behalf of citizens and confidence in collective decision-making and deliberation processes. A society with high levels of democratic political culture may be conducive to actively accepting and sharing its governments' decisions to combat COVID-19 facilitating successful governance.

Government function (−203.14), civil liberties (−149.23) and electoral process and pluralism (−75.64) are also associated with lower levels of death. It is intuitive that better government management capacity means better execution of health policies when addressing the pandemic and this is consistent with studies which have outlined that better public governance is shown to be related to better indicators of public governance management^3^. Work by Boris and Pellizo^47^, Liang et al.^48^ and Bunyavejchewin and Sirichuanjun^4^ link improved government effectiveness to reduced COVID-19 mortality. Civil liberties and electoral process and pluralism consisting of 9 and 12 items, respectively, is defined as the space of individual rights, freedom of expression and opinion. They refer directly to transparency in public deliberation and the right to dissent. Societies with high levels of civil liberties and pluralism are better prepared to reject opportunistic political responses and misinformation and to make better informed collective decisions in uncertain environments such as the one generated by the pandemic.

In any case, the strong interrelationship and feedback of all the dimensions which make up the democracy index should not be forgotten.

Positive relationships between democratic quality and health system outcomes are not necessarily limited to the pandemic era. Several studies have found robust associations between higher democratic quality and better health outcomes in the past^49,50^. Bousmah et al.^51^ for example find that increased health spending leads to improvements in health outcomes only to the extent that the quality of institutions within a country is sufficiently high. Our results indicate that the virtuous circle between democratic quality and health outcomes is maintained in times of global public health crises showing greater resilience and responsiveness to disruptive crises such as Covid-19.

The paper has several limitations. Although the excess mortality more adequately captures the impact of COVID-19 mortality than the mortality rate, it does not capture all the suffering and illness created by the pandemic. In addition to fatalities Coronavirus has caused several waves of morbidity which governments will need to address in the future: postponed chronic patients and new chronic COVID-19 sufferers^52^, mental health^53,54^ and persistent/long COVID-19^55^.

The reporting and registry system providing data on excess mortality is complex and requires adequate infrastructure. Many countries do not have such infrastructure and consequently the number of countries with available data is limited. This restriction may have led to selection bias and reduced the power of the estimates. However, in terms of opportunity cost it is worthwhile compared to studies with a larger number of countries, but with more limited measurements of COVID-19 mortality.

Finally, an omission bias in the control variables employed is possible, despite the careful scrutiny carried out. We preferred to use a parsimony criterion in the specification of the models based on the variables which offered the highest level of consensus in the literature. However, future research should address dimensions which were not considered in detail in this study. For example, a more detailed scrutiny of the relevance of income and wealth inequality in each country, its relationship with democratic quality, and the effectiveness of pandemic management.

More than two decades ago, Amartya Sen^56^ stated in his 1999 book "Development as freedom" that "No famine has ever taken place in the history of the world in a functional democracy". Going against the efficient autocracies hypothesis this paper finds strong evidence that in Sen's terminology "functional democracies", i.e., those with high levels of democratic quality also protect from death from COVID-19 better than countries with democratic deficits. The result is useful in the current debate on the relationship between political regimes and pandemic management, suggesting that higher democratic quality is an effective public health policy in the face of Coronavirus.

## Data Availability

The datasets generated and/or analysed that support the findings of this study are available in the links of the different repositories, in Table 1, also they are available from the corresponding author upon request.

## Annex I: Democracy indices: indicators and items by categories

**Table:**

Categories/dimensions	Items
Category I (electoral process and pluralism)	1. Existence of free national elections 2. Existence of fair national elections 3. Existence of free and fair municipal elections 4. Universal suffrage 5. Citizens can vote without significant threats to their security 6. Laws provide equal opportunity to conduct election campaigns 7. Political party financing is transparent and generally accepted 8. After elections, constitutional mechanisms for the orderly transfer of power are clear, established and accepted 9. Freedom of citizens to form political parties independent of government 10. Opposition parties have a realistic chance of reaching government 11. Access to public office is open to all citizens 12. Citizens can form civic and political organisations free from state interference and surveillance
Category II (government function)	13. Freely elected representatives determine government policy 14. Supremacy of the legislature over all other branches of government 15. Existence of an effective system of checks and balances in the exercise of governmental authority 16. The government is free from undue interference by the military or security services 17. Foreign powers and organisations do not determine important government functions or policies 18. Economic, religious or other domestically powerful groups do not exercise significant political power in parallel to democratic institutions 19. Sufficient mechanisms and institutions exist to ensure government accountability to the electorate in the period between elections 20. Government authority extends over the entire territory of the country 21. The functioning of government is open and transparent with sufficient public access to information 22. Corruption is not widespread 23. The public administration is willing and able to implement government policy 24. Popular perception of the degree to which citizens have freedom of choice and control over their lives 25. Citizens' trust in government 26. Citizens' trust in political parties
Category III (political participation)	27. Average turnout in elections since 2000 (over 70%) 28. Ethnic, religious and other minorities have a reasonable degree of autonomy and voice in the political process 29. More than 20 per cent of parliamentarians are women 30. Degree of political participation (over 7% of the population is a member of political parties and organisations) 31. Degree of citizen engagement in politics 32. The preparedness of population to take part in lawful demonstrations 33. Adult literacy rate (> 90%) 34. Extent to which the adult population shows interest and follows politics in the news 35. Authorities make a serious effort to promote political participation
Category IV (democratic political culture)	36. The existence of a sufficient degree of social consensus and cohesion to maintain a stable and functioning democracy 37. Proportion of the population that does not desire a strong leader who bypasses parliament and elections 38. Proportion of the population that would prefer a military government (< 10%) 39. Proportion of the population that would prefer a government of experts or technocrats (< 50%) 40. Percentage of population who believe that democracies are not good at ensuring law and order (< 50%) 41. Percentage of population who believe that democracy benefits a country's economy (> 80%) 42. Degree of popular support for democracy 43. Strong tradition of separation of church and state
Category V (civil liberties)	44. Existence of free electronic means of communication 45. Existence of freedom of the press 46. Existence of freedom of expression and protest 47. The media coverage is robust. There is an open and free discussion of public issues, with a reasonable diversity of opinions 48. There are no political restrictions on Internet access 49. Citizens are free to form professional organisations and trade unions 50. The institutions provide citizens with the opportunity to petition government to redress grievances 51. Torture is not used by the state 52. Independence of the judiciary from government influence 53. The degree of religious tolerance and freedom of religious expression 54. Equality of all citizens before the law 55. Security of citizens 56. Degree of protection of private property rights and absence of undue government influence on private business 57. Extent to which citizens enjoy personal freedoms 58. Popular perception of the protection of human rights 59. Absence of significant discrimination on the basis of race, colour or religious belief 60. Extent to which the government invokes new risks and threats as an excuse to restrict civil liberties

Adapted from The Economist Intelligence Unit Limited^7^. https://www.eiu.com/n/campaigns/democracy-index-2020/.

## Annex II Excess mortality, overall democracy index by categories for 80 countries. 2020.

**Table:**

	Excess mortality per million inhabitants	Overall democracy Index	Category 1	Category 2	Category 3	Category 4	Category 5
Africa							
Mauritius	−302.18	8.14	9.17	7.86	6.11	8.75	8.82
South Africa	994.62	7.05	7.42	7.14	8.33	5	7.35
Tunisia	212.09	6.59	9.17	5.36	7.22	5.63	5.59
América/Caribe							
Argentina	919.80	6.95	9.17	5.36	6.67	5.63	7.94
Bolivia	1911.17	5.08	6.08	3.57	6.11	3.75	5.88
Brazil	1016.66	6.92	9.58	5.36	6.11	5.63	7.94
Canada	474.28	9.24	9.58	8.93	8.89	9.38	9.41
Chile	763.39	8.28	9.58	8.21	6.67	8.13	8.82
Colombia	983.15	7.04	9.17	6.43	6.67	5	7.94
Costa Rica	179.18	8.16	9.58	6.79	7.22	7.5	9.71
Ecuador	2211.62	6.13	8.75	5	6.67	3.75	6.47
El Salvador	727.01	5.9	9.17	4.29	6.11	3.75	6.18
Guatemala	606.57	4.97	6.92	3.93	5	3.13	5.88
Jamaica	−221.90	7.13	8.75	7.14	5	6.25	8.53
México	2304.86	6.07	7.83	5.71	7.78	3.13	5.88
Nicaragua	1017.46	3.6	0.42	2.86	5	5.63	4.12
Panama	688.24	7.18	9.58	6.43	7.22	5	7.65
Paraguay	343.84	6.18	8.75	5.71	5	4.38	7.06
Peru	2626.40	6.53	8.75	5.36	5.56	5.63	7.35
United States	1417.91	7.92	9.17	6.79	8.89	6.25	8.53
Uruguay	−657.76	8.61	10	8.57	6.67	8.13	9.71
Asia							
Armenia	3377.21	5.35	7.5	5	6.11	3.13	5
Egypt	1012.68	2.93	1.33	3.21	3.33	5	1.76
Georgia	1283.79	5.31	7.83	3.57	6.11	3.75	5.29
Israel	317.02	7.84	9.17	7.5	9.44	7.5	5.59
Iran	1354.96	2.2	0	2.5	3.89	3.13	1.47
Japan	−222.16	8.13	8.75	8.57	6.67	8.13	8.53
Kazakhstan	1586.73	3.14	0.5	3.21	5	3.75	3.24
Kirgizstan	1128.82	4.21	4.75	2.93	5.56	3.13	4.71
Lebanon	438.28	4.16	3.5	1.5	6.67	5	4.12
Malaysia	−276.90	7.19	9.58	7.86	6.67	6.25	5.59
Mongolia	−451.21	6.48	8.75	5.71	5.56	5.63	6.76
Oman	244.90	3	0.08	3.93	2.78	4.38	3.82
Philippines	−98.81	6.56	9.17	5	7.78	4.38	6.47
Qatar	128.71	3.24	0	4.29	2.78	5.63	3.53
Russia	2457.01	3.31	2.17	2.14	5	3.13	4.12
Singapore	3.73	6.03	4.83	7.86	4.44	6.25	6.76
South Korea	3.33	8.01	9.17	8.21	7.22	7.5	7.94
Thailand	48.26	6.04	7	5	6.67	6.25	5.29
Uzbekistan	664.23	2.12	0.08	1.86	2.78	5	0.88
Europa							
Albania	2023.16	6.08	7	5.36	4.44	6.25	7.35
Azerbaijan	1878.05	2.68	0.5	2.86	3.33	3.75	2.94
Austria	859.62	8.16	9.58	7.5	8.33	6.88	8.53
Belarus	2527.73	2.59	0	2	3.89	5	2.06
Belgium	1486.49	7.51	9.58	7.86	5	6.88	8.24
Bosnia and Herzegovina	2021.11	4.84	7	2.93	5.56	3.13	5.59
Bulgaria	2436.09	6.71	9.17	5.71	7.22	4.38	7.06
Croatia	1312.92	6.5	9.17	6.07	6.11	4.38	6.76
Cyprus	62.84	7.56	9.17	5.36	7.22	7.5	8.53
Czechia	1512.27	7.67	9.58	6.07	6.67	7.5	8.53
Denmark	−99.60	9.15	10	8.93	8.33	9.38	9.12
Estonia	115.68	7.84	9.58	7.86	6.67	6.88	8.24
Finlandia	74.87	9.2	10	8.93	8.89	8.75	9.41
France	701.00	7.99	9.58	7.5	7.78	6.88	8.24
Germany	371.98	8.67	9.58	8.21	8.33	8.13	9.12
Greece	580.38	7.39	9.58	5.21	6.11	7.5	8.53
Hungary	1118.14	6.56	8.33	6.43	5	6.25	6.76
Iceland	55.04	9.37	10	8.57	8.89	10	9.41
Ireland	28.42	9.05	10	7.86	8.33	9.38	9.71
Italia	1688.21	7.74	9.58	6.43	7.22	7.5	7.94
Latvia	390.69	7.24	9.58	6.07	6.67	5.63	8.24
Lithuania	2236.14	7.13	9.58	6.07	5.56	5.63	8.82
Malta	424.05	7.68	9.17	6.79	6.11	8.13	8.24
Moldova	1262.82	5.78	7	4.64	6.11	4.38	6.76
Montenegro	1063.29	5.77	7.42	5.71	6.11	3.13	6.47
Netherlands	819.44	8.96	9.58	9.29	8.33	8.75	8.82
Norway	−42.52	9.81	10	9.64	10	10	9.41
Polonia	1615.87	6.85	9.17	5.71	6.67	5.63	7.06
Portugal	990.68	7.9	9.58	7.5	6.11	7.5	8.82
Rumania	1857.51	6.4	9.17	5.36	6.67	3.75	7.06
Serbia	2335.86	6.22	8.25	5.36	6.67	3.75	7.06
Slovakia	1024.06	6.97	9.58	6.43	5.56	5.63	7.65
Slovenia	1497.70	7.54	9.58	6.43	7.22	6.25	8.24
Spain	1477.24	8.12	9.58	7.14	7.22	8.13	8.53
Sweden	786.98	9.26	9.58	9.29	8.33	10	9.12
Switzerland	970.16	8.83	9.58	8.57	7.78	9.38	8.82
United Kingdom	1171.61	8.54	10	7.5	8.89	7.5	8.82
Ukraine	910.30	5.81	8.25	2.71	7.22	5	5.88
Oceania							
Australia	−134.29	8.96	10	8.57	7.78	8.75	9.71
New Zealand	−445.13	9.25	10	8.93	8.89	8.75	9.71

Author’s own using Our World in Data (2022) https://ourworldindata.org/, The Economist Intelligence Unit Limited^7^. Democracy Index 2020. In sickness and in health? https://www.eiu.com/n/campaigns/democracy-index-2020/ Last used: 01/07/2021.
